# Supplementary material for: Childhood trauma and schizotypy in non-clinical samples: A systematic review and meta-analysis
Source: PLoS One. 2022 Jun 29;17(6):e0270494. doi: 10.1371/journal.pone.0270494 (PMC9242513; doi:10.1371/journal.pone.0270494)
Supplement: S1 Appendix — (DOCX) [file pone.0270494.s001.docx]

Appendix - References (screened-excluded)

Addington, J., Stowkowy, J., Cadenhead, K. S., Cornblatt, B. A., McGlashan, T. H., Perkins, D. O., Seidman, L. J., Tsuang, M. T., Walker, E. F., Woods, S. W., & Cannon, T. D. (2013). Early traumatic experiences in those at clinical high risk for psychosis. *Early Intervention in Psychiatry, 7*(3), 300-305. doi: 10.1111/eip.12020

Afifi, T. O., Mather, A., Boman, J., Fleisher, W., Enns, M. W., MacMillan, H., & Sareen, J. (2011). Childhood adversity and personality disorders: Results from a nationally representative population-based study. *Journal of Psychiatric Research, 45*(6), 814-822. doi: 10.1016/j.jpsychires.2010.11.008

Airey, N. D., Hammersley, R., & Reid, M. (2020). Schizotypy but not Cannabis Use Modestly Predicts Psychotogenic Experiences: A Cross-Sectional Study Using the Oxford-Liverpool Inventory of Feelings and Experiences (O-LIFE). *J Addict, 2020*, 5961275. doi: 10.1155/2020/5961275

Alemany, S., Arias, B., Aguilera, M., Villa, H., Moya, J., Ibañez, M. I., Vossen, H., Gasto, C., Ortet, G., & Fañanas, L. (2011). P03-212 - Childhood Abuse and the BDNF-Val66Met Polymorphism: Evidence for Gene-Environment Interaction in the Development of Adult Psychosis-Like Experiences. *European Psychiatry, 26*, 1381-1381. doi: 10.1016/S0924-9338(11)73086-3

Ayazi, T., Swartz, L., Eide, A. H., Lien, L., & Hauff, E. (2016). Psychotic-like experiences in a conflict-affected population: a cross-sectional study in South Sudan. *Soc Psychiatry Psychiatr Epidemiol, 51*(7), 971-979. doi: 10.1007/s00127-016-1243-2

Barrantes-Vidal, N., Lewandowski, K. E., & Kwapil, T. R. (2010). Psychopathology, social adjustment and personality correlates of schizotypy clusters in a large nonclinical sample. *Schizophrenia research, 122*(1-3), 219-225.

Battle, C. L., Shea, M. T., Johnson, D. M., Yen, S., Zlotnick, C., Zanarini, M. C., Sanislow, C. A., Skodol, A. E., Gunderson, J. G., Grilo, C. M., McGlashan, T. H., & Morey, L. C. (2004). Childhood maltreatment associated with adult personality disorders: findings from the Collaborative Longitudinal Personality Disorders Study. *Journal of personality disorders, 18*(2), 193-211.

Bechdolf, A., Thompson, A., Nelson, B., Cotton, S., Simmons, M. B., Amminger, G. P., Leicester, S., Francey, S. M., McNab, C., Krstev, H., Sidis, A., McGorry, P. D., & Yung, A. R. (2010). Experience of trauma and conversion to psychosis in an ultra-high-risk (prodromal) group. *Acta psychiatrica Scandinavica, 121*(5), 377-384.

Berenz, E. C., Amstadter, A. B., Aggen, S. H., Peggy Knudsen, G., Reichborn-Kjennerud, T., Gardner, C. O., & Kendler, K. S. (2013). Childhood Trauma and Personality Disorder Criterion Counts: A Co-twin Control Analysis. *Journal of Abnormal Psychology, 122*(4), 1070-1076. doi: 10.1037/a0034238

Berkowski, M., & MacDonald, D. A. (2014). Childhood trauma and the development of paranormal beliefs. *The Journal of nervous and mental disease, 202*(4), 305-312.

Berry, K., Band, R., Corcoran, R., Barrowclough, C., & Wearden, A. (2007). Attachment styles, earlier interpersonal relationships and schizotypy in a non-clinical sample. *Psychology & Psychotherapy: Theory, Research & Practice, 80*(4), 563-576. doi: 10.1348/147608307X188368

Binbay, T., Drukker, M., Elbi, H., Tanik, F. A., Ozkinay, F., Onay, H., Zagli, N., van Os, J., & Alptekin, K. (2012). Testing the psychosis continuum: differential impact of genetic and nongenetic risk factors and comorbid psychopathology across the entire spectrum of psychosis. *Schizophrenia bulletin, 38*(5), 992-1002.

Boden, J. M., van Stockum, S., Horwood, L. J., & Fergusson, D. M. (2016). Bullying victimization in adolescence and psychotic symptomatology in adulthood: evidence from a 35-year study. *Psychological Medicine, 46*(6), 1311-1320. doi: 10.1017/S0033291715002962

Boyda, D., & McFeeters, D. (2015). Childhood maltreatment and social functioning in adults with sub-clinical psychosis. *Psychiatry Research, 226*(1), 376-382. doi: 10.1016/j.psychres.2015.01.023

Carvalho, C. B., da Motta, C., Pinto-Gouveia, J., & Peixoto, E. (2018). Psychosocial roots of paranoid ideation: The role of childhood experiences, social comparison, submission, and shame. *Clin Psychol Psychother, 25*(5), 650-661. doi: 10.1002/cpp.2195

Catone, G., Marwaha, S., Kuipers, E., Lennox, B., Freeman, D., Bebbington, P., & Broome, M. (2015). Bullying victimisation and risk of psychotic phenomena: analyses of British national survey data. *The lancet. Psychiatry, 2*(7), 618-624.

Colins, O., Vermeiren, R., Vreugdenhil, C., Schuyten, G., Broekaert, E., & Krabbendam, A. (2009). Are psychotic experiences among detained juvenile offenders explained by trauma and substance use? *Drug and alcohol dependence, 100*(1-2), 39-46.

Coughlan, H., Healy, C., Sheaghdha, A. N., Murray, G., Humphries, N., Clarke, M., & Cannon, M. (2020). Early risk and protective factors and young adult outcomes in a longitudinal sample of young people with a history of psychotic-like experiences. *Early Intervention in Psychiatry, 14*(3), 307-320. doi: 10.1111/eip.12855

Cristóbal-Narváez, P., Sheinbaum, T., Rosa, A., Ballespí, S., de Castro-Catala, M., Peña, E., Kwapil, T. R., & Barrantes-Vidal, N. (2016). The Interaction between Childhood Bullying and the FKBP5 Gene on Psychotic-Like Experiences and Stress Reactivity in Real Life. *PLoS ONE, 11*(7), 1-14. doi: 10.1371/journal.pone.0158809

de Leede-Smith, S., & Barkus, E. (2013). A comprehensive review of auditory verbal hallucinations: lifetime prevalence, correlates and mechanisms in healthy and clinical individuals. *Frontiers in human neuroscience, 7*, 367-367.

Dias, A., Sales, L., Hessen, D., & Kleber, R. (2015). Child maltreatment and psychological symptoms in a Portuguese adult community sample: the harmful effects of emotional abuse. *European Child & Adolescent Psychiatry, 24*(7), 767-778. doi: 10.1007/s00787-014-0621-0

Diaz, M. A., Dickerson, L. A., & Kwapil, T. R. (2005). Life events and perceived stress in schizotypic young adults: A three-year follow-up study. *Schizophrenia Bulletin, 31*(2), 354-354.

Dickson, J. M., Barsky, J., Kinderman, P., King, D., & Taylor, P. J. (2016). Early relationships and paranoia: Qualitative investigation of childhood experiences associated with the development of persecutory delusions. *Psychiatry research, 238*, 40-45.

Fisher, H. L., Schreier, A., Zammit, S., Maughan, B., Munafo, M. R., Lewis, G., & Wolke, D. (2013). Pathways between childhood victimization and psychosis-like symptoms in the ALSPAC birth cohort. *Schizophrenia bulletin, 39*(5), 1045-1055.

Frydecka, D., Kotowicz, K., Gaweda, L., Prochwicz, K., Klosowska, J., Rymaszewska, J., Samochowiec, A., Samochowiec, J., Podwalski, P., Pawlak-Adamska, E., Szmida, E., Cechnicki, A., & Misiak, B. (2020). Effects of interactions between variation in dopaminergic genes, traumatic life events, and anomalous self-experiences on psychosis proneness: Results from a cross-sectional study in a nonclinical sample. *Eur Psychiatry, 63*(1), e104. doi: 10.1192/j.eurpsy.2020.103

Frydecka, D., Misiak, B., Kotowicz, K., Pionke, R., Krezolek, M., Cechnicki, A., & Gaweda, L. (2020). The interplay between childhood trauma, cognitive biases, and cannabis use on the risk of psychosis in nonclinical young adults in Poland. *Eur Psychiatry, 63*(1), e35. doi: 10.1192/j.eurpsy.2020.31

Gaweda, L., Pionke, R., Krezolek, M., Frydecka, D., Nelson, B., & Cechnicki, A. (2020). The interplay between childhood trauma, cognitive biases, psychotic-like experiences and depression and their additive impact on predicting lifetime suicidal behavior in young adults. *Psychol Med, 50*(1), 116-124. doi: 10.1017/S0033291718004026

Goldstone, E., Farhall, J., & Ong, B. (2011). Life hassles, experiential avoidance and distressing delusional experiences. *Behaviour research and therapy, 49*(4), 260-266.

Gong, J., Wang, Y., Liu, J., Fu, X., Cheung, E. F. C., & Chan, R. C. K. (2019). The interaction between positive schizotypy and high sensitivity C-reactive protein on response inhibition in female individuals. *Psychiatry Res, 274*, 365-371. doi: 10.1016/j.psychres.2019.02.064

Gurian, B. S., Wexler, D., & Baker, E. H. (1992). LATE-LIFE PARANOIA: POSSIBLE ASSOCIATION WITH EARLY TRAUMA AND INFERTILITY. *International Journal of Geriatric Psychiatry, 7*(4), 277-284. doi: 10.1002/gps.930070409

Heins, M., Simons, C., Lataster, T., Pfeifer, S., Versmissen, D., Lardinois, M., Marcelis, M., Delespaul, P., Krabbendam, L., van Os, J., & Myin-Germeys, I. (2011). Childhood Trauma and Psychosis: A Case-Control and Case-Sibling Comparison Across Different Levels of Genetic Liability, Psychopathology, and Type of Trauma. *American Journal of Psychiatry, 168*(12), 1286-1294. doi: 10.1176/appi.ajp.2011.10101531

Humpston, C. S., Walsh, E., Oakley, D. A., Mehta, M. A., Bell, V., & Deeley, Q. (2016). The relationship between different types of dissociation and psychosis-like experiences in a non-clinical sample. *Consciousness and cognition, 41*, 83-92.

Johnson, J. G., Cohen, P., Smailes, E. M., Skodol, A. E., Brown, J., & Oldham, J. M. (2001). Childhood verbal abuse and risk for personality disorders during adolescence and early adulthood. *Comprehensive Psychiatry, 42*(1), 16-23.

Johnson, J. G., Smailes, E. M., Cohen, P., Brown, J., & Bernstein, D. P. (2000). Associations between four types of childhood neglect and personality disorder symptoms during adolescence and early adulthood: findings of a community-based longitudinal study. *Journal of personality disorders, 14*(2), 171-187.

Kelleher, I., Wigman, J. T. W., Harley, M., O'Hanlon, E., Coughlan, H., Rawdon, C., Murphy, J., Power, E., Higgins, N. M., & Cannon, M. (2015). Psychotic experiences in the population: Association with functioning and mental distress. *Schizophrenia research, 165*(1), 9-14.

Kelly, J., & Murray, R. M. (2000). What risk factors tell us about the causes of schizophrenia and related psychoses. *Current psychiatry reports, 2*(5), 378-385.

Kerns, J. G. (2005). Positive schizotypy and emotion processing. *Journal of Abnormal Psychology, 114*(3), 392-401.

Kilcommons, A. M., Morrison, A. P., Knight, A., & Lobban, F. (2008). Psychotic experiences in people who have been sexually assaulted. *Social Psychiatry & Psychiatric Epidemiology, 43*(8), 602-611. doi: 10.1007/s00127-007-0303-z

Kingston, C., & Schuurmans-Stekhoven, J. (2016). Life hassles and delusional ideation: Scoping the potential role of cognitive and affective mediators. *Psychology and psychotherapy, 89*(4), 445-463.

Kline, E., Millman, Z. B., Denenny, D., Wilson, C., Thompson, E., Demro, C., Connors, K., Bussell, K., Reeves, G., & Schiffman, J. (2016). Trauma and psychosis symptoms in a sample of help-seeking youth. *Schizophrenia Research, 175*(1-3), 174-179. doi: 10.1016/j.schres.2016.04.006

Kocsis-Bogar, K., Miklosi, M., & Forintos, D. P. (2013). Impact of adverse life events on individuals with low and high schizotypy in a nonpatient sample. *The Journal of nervous and mental disease, 201*(3), 208-215.

Koyanagi, A., Stickley, A., & Haro, J. M. (2015). Psychotic-Like Experiences and Nonsuidical Self-Injury in England: Results from a National Survey. *PLoS ONE, 10*(12), 1-12. doi: 10.1371/journal.pone.0145533

Laloyaux, J., Dessart, G., Van der Linden, M., Lemaire, M., & Laroi, F. (2016). Maladaptive emotion regulation strategies and stress sensitivity mediate the relation between adverse life events and attenuated positive psychotic symptoms. *Cognitive neuropsychiatry, 21*(2), 116-129.

Langer, A. I., Stanghellini, G., Cangas, A. J., Lysaker, P. H., Nieto-Munoz, L., Moriana, J. A., Barrigon, M. L., & Ambrosini, A. (2015). Interpretation, emotional reactions and daily life implications of hallucination-like experiences in clinical and nonclinical populations. *Psicothema, 27*(1), 19-25.

Lataster, T., van Os, J., Drukker, M., Henquet, C., Feron, F., Gunther, N., & Myin-Germeys, I. (2006). Childhood victimisation and developmental expression of non-clinical delusional ideation and hallucinatory experiences - Victimisation and non-clinical Psychotic experiences. *Social Psychiatry and Psychiatric Epidemiology, 41*(6), 423-428. doi: 10.1007/s00127-006-0060-4

Leiderman, E. A. (2011). Psychotic-like experiences in the general population of Buenos Aires city. *Schizophr Res, 130*(1-3), 291-292. doi: 10.1016/j.schres.2011.03.009

Liu, J., Gong, J., Nie, G., He, Y., Xiao, B., Shen, Y., & Luo, X. (2017). The mediating effects of childhood neglect on the association between schizotypal and autistic personality traits and depression in a non-clinical sample. *BMC Psychiatry, 17*(1), 352. doi: 10.1186/s12888-017-1510-0

Lobbestael, J., Arntz, A., & Bernstein, D. P. (2010). DISENTANGLING THE RELATIONSHIP BETWEEN DIFFERENT TYPES OF CHILDHOOD MALTREATMENT AND PERSONALITY DISORDERS. *Journal of Personality Disorders, 24*(3), 285-295. doi: 10.1521/pedi.2010.24.3.285

Lopes, B. C. (2013). Differences Between Victims of Bullying and Nonvictims on Levels of Paranoid Ideation and Persecutory Symptoms, the Presence of Aggressive Traits, the Display of Social Anxiety and the Recall of Childhood Abuse Experiences in a Portuguese Mixed Clinical Sample. *Clinical Psychology & Psychotherapy, 20*(3), 254-266. doi: 10.1002/cpp.800

Lovatt, A., Mason, O., Brett, C., & Peters, E. (2010). Psychotic-like experiences, appraisals, and trauma. *The Journal of nervous and mental disease, 198*(11), 813-819.

Mackie, C. J., Castellanos-Ryan, N., & Conrod, P. J. (2011). Developmental trajectories of psychotic-like experiences across adolescence: impact of victimization and substance use. *Psychological Medicine, 41*(1), 47-58. doi: 10.1017/S0033291710000449

Marzillier, S. L., & Steel, C. (2007). Positive schizotypy and trauma-related intrusions. *Journal of Nervous and Mental Disease, 195*(1), 60-64.

Memis, C. O., Dogan, B., Sevincok, D., Ashik, I., & Sevincok, L. (2020). Mediating role of childhood abuse for the relationship between schizotypal traits and obsessive-compulsive disorder. *Archives of Clinical Psychiatry (São Paulo), 47*(2), 40-44. doi: 10.1590/0101-60830000000229

Mojtabai, R. (2006). Psychotic-like experiences and interpersonal violence in the general population. *Social psychiatry and psychiatric epidemiology, 41*(3), 183-190.

Murphy, J., Shevlin, M., Adamson, G., Cruddas, S., & Houston, J. (2012). Memories of Childhood Threat, Fear of Disclosure and Paranoid Ideation: A Mediation Analysis Using a Nonclinical Sample. *Journal of Aggression, Maltreatment & Trauma, 21*(4), 459-476. doi: 10.1080/10926771.2012.667521

Murphy, J., Shevlin, M., Houston, J., & Adamson, G. (2014). Modelling the co-occurrence of psychosis-like experiences and childhood sexual abuse. *Social Psychiatry & Psychiatric Epidemiology, 49*(7), 1037-1044. doi: 10.1007/s00127-014-0845-9

Narita, Z., Stickley, A., & DeVylder, J. (2020). Loneliness and psychotic experiences in a general population sample. *Schizophr Res, 218*, 146-150. doi: 10.1016/j.schres.2020.01.018

Oh, H., Cogburn, C. D., Anglin, D., Lukens, E., & DeVylder, J. (2016). Major discriminatory events and risk for psychotic experiences among Black Americans. *The American journal of orthopsychiatry, 86*(3), 277-285.

Oh, H., Yang, L. H., Anglin, D. M., & DeVylder, J. E. (2014). Perceived discrimination and psychotic experiences across multiple ethnic groups in the United States. *Schizophrenia research, 157*(1-3), 259-265.

Parker, G., Roy, K., Wilhelm, K., Mitchell, P., Austin, M. P., & Hadzi-Pavlovic, D. (1999). An exploration of links between early parenting experiences and personality disorder type and disordered personality functioning. *J Pers Disord, 13*(4), 361-374. doi: 10.1521/pedi.1999.13.4.361

Peh, O. H., Rapisarda, A., & Lee, J. (2020). Quality of parental bonding is associated with symptom severity and functioning among individuals at ultra-high risk for psychosis. *Schizophrenia Research, 215*, 204-210. doi: 10.1016/j.schres.2019.10.029

Peters, E., Ward, T., Jackson, M., Morgan, C., Charalambides, M., McGuire, P., Woodruff, P., Jacobsen, P., Chadwick, P., & Garety, P. A. (2016). Clinical, socio-demographic and psychological characteristics in individuals with persistent psychotic experiences with and without a "need for care". *World Psychiatry, 15*(1), 41-52. doi: 10.1002/wps.20301

Pionke, R., Gidzgier, P., Nelson, B., & Gawęda, Ł. (2020). Prevalence, dimensionality and clinical relevance of self‐disturbances and psychotic‐like experiences in Polish young adults: a latent class analysis approach. *International Journal of Methods in Psychiatric Research, 29*(1), 1-11. doi: 10.1002/mpr.1809

Rossi, R., Collazzoni, A., Talevi, D., Gibertoni, D., Quarta, E., Rossi, A., Stratta, P., Di Lorenzo, G., & Pacitti, F. (2020). Personal and contextual components of resilience mediate risky family environment's effect on psychotic-like experiences. *Early Interv Psychiatry*. doi: 10.1111/eip.13111

Rossi, R., Collazzoni, A., Talevi, D., Quarta, E., Gregori, E., Rossi, A., Stratta, P., Pacitti, F., & Di Lorenzo, G. (2020). Personal and contextual components of resilience mediate childhood adversity's effect on psychotic-like experiences. *European Psychiatry, 63*, S211-S211.

Rossi, R., Socci, V., Collazzoni, A., Lucaselli, A., Di Lorenzo, G., & Pacitti, F. (2019). Psychotic-like experiences interaction with common risk factors for suicidal ideation. *Journal of Psychopathology, 25*(4), 205-211.

Rössler, W., Ajdacic-Gross, V., Rodgers, S., Haker, H., & Müller, M. (2016). Childhood trauma as a risk factor for the onset of subclinical psychotic experiences: Exploring the mediating effect of stress sensitivity in a cross-sectional epidemiological community study. *Schizophrenia Research, 172*(1-3), 46-53. doi: 10.1016/j.schres.2016.02.006

Rössler, W., Vetter, S., Müller, M., Gallo, W. T., Haker, H., Kawohl, W., Lupi, G., & Ajdacic-Gross, V. (2011). Risk factors at the low end of the psychosis continuum: Much the same as at the upper end? *Psychiatry Research, 189*(1), 77-81. doi: 10.1016/j.psychres.2011.02.019

Russo, D. A., Stochl, J., Painter, M., Dobler, V., Jackson, E., Jones, P. B., & Perez, J. (2014). Trauma history characteristics associated with mental states at clinical high risk for psychosis. *Psychiatry Res, 220*(1-2), 237-244. doi: 10.1016/j.psychres.2014.08.028

Saha, S., Varghese, D., Slade, T., Degenhardt, L., Mills, K., McGrath, J., & Scott, J. (2011). The association between trauma and delusional-like experiences. *Psychiatry Research, 189*(2), 259-264. doi: 10.1016/j.psychres.2011.03.019

Schoorl, J., Barbu, M. C., Shen, X., Harris, M. R., Adams, M. J., Whalley, H. C., & Lawrie, S. M. (2021). Grey and white matter associations of psychotic-like experiences in a general population sample (UK Biobank). *Transl Psychiatry, 11*(1), 21. doi: 10.1038/s41398-020-01131-7

Schurhoff, F., Laguerre, A., Fisher, H., Etain, B., Meary, A., Soussy, C., Szoke, A., & Leboyer, M. (2009). Self-reported childhood trauma correlates with schizotypal measures in schizophrenia but not bipolar pedigrees. *Psychological medicine, 39*(3), 365-370.

Sengutta, M., Gawęda, Ł., Moritz, S., & Karow, A. (2019). The mediating role of borderline personality features in the relationship between childhood trauma and psychotic-like experiences in a sample of help-seeking non-psychotic adolescents and young adults. *European Psychiatry, 56*, 84-90. doi: 10.1016/j.eurpsy.2018.11.009

Sharifi, V., Bakhshaie, J., Hatmi, Z., Faghih-Nasiri, L., Sadeghianmehr, Z., Mirkia, S., Darbooy, S., Effatpanah, M., & Mirsharifa, S. M. (2012). Self-Reported Psychotic Symptoms in the General Population: Correlates in an Iranian Urban Area. *Psychopathology, 45*(6), 374-380. doi: 10.1159/000337749

Sheinbaum, T., Bedoya, E., Ros-Morente, A., Kwapil, T. R., & Barrantes-Vidal, N. (2013). Association between attachment prototypes and schizotypy dimensions in two independent non-clinical samples of Spanish and American young adults. *Psychiatry Research, 210*(2), 408-413. doi: 10.1016/j.psychres.2013.07.020

Sheinbaum, T., Bifulco, A., Ballespí, S., Mitjavila, M., Kwapil, T. R., & Barrantes-Vidal, N. (2015). Interview Investigation of Insecure Attachment Styles as Mediators between Poor Childhood Care and Schizophrenia-Spectrum Phenomenology. *PLoS ONE, 10*(8), 1-12. doi: 10.1371/journal.pone.0135150

Sheinbaum, T., Racioppi, A., Kwapil, T. R., & Barrantes-Vidal, N. (2020). Attachment as a mechanism between childhood maltreatment and subclinical psychotic phenomena: Results from an eight-year follow-up study. *Schizophrenia Research, 220*, 261-264. doi: 10.1016/j.schres.2020.03.023

Shevlin, M., O'Neill, T., Houston, J. E., Read, J., Bentall, R. P., & Murphy, J. (2013). Patterns of lifetime female victimisation and psychotic experiences: a study based on the UK Adult Psychiatric Morbidity Survey 2007. *Social psychiatry and psychiatric epidemiology, 48*(1), 15-24.

Sommer, I. E. C., Daalman, K., Rietkerk, T., Diederen, K. M., Bakker, S., Wijkstra, J., & Boks, M. P. M. (2010). Healthy individuals with auditory verbal hallucinations; who are they? Psychiatric assessments of a selected sample of 103 subjects. *Schizophrenia bulletin, 36*(3), 633-641.

Strelchuk, D., Hammerton, G., Croft, J., Heron, J., Zammit, S., Wiles, N., & Turner, K. (2020). PTSD AS A MEDIATOR OF THE RELATIONSHIP BETWEEN TRAUMA AND PSYCHOTIC EXPERIENCES. *Schizophrenia Bulletin, 46*, S273-S273.

Sun, M., Hu, X., Zhang, W., Guo, R., Hu, A., Mwansisya, T. E., Zhou, L., Liu, C., Chen, X., Huang, X., Shi, J., Chiu, H. F. K., & Liu, Z. (2015). Psychotic-like experiences and associated socio-demographic factors among adolescents in China. *Schizophrenia Research, 166*(1-3), 49-54. doi: 10.1016/j.schres.2015.05.031

Sun, M., Xue, Z., Zhang, W., Guo, R., Hu, A., Li, Y., Mwansisya, T. E., Zhou, L., Liu, C., Chen, X., Huang, X., Tao, H., Shi, J., Liu, Z., & Rosenheck, R. (2017). Psychotic-like experiences, trauma and related risk factors among “left-behind” children in China. *Schizophrenia Research, 181*, 43-48. doi: 10.1016/j.schres.2016.09.030

Sun, M., Zhang, W., Guo, R., Hu, A., Li, Y., Mwansisya, T. E., Zhou, L., Liu, C., Chen, X., Tao, H., Huang, X., Xue, Z., Chiu, H. F. K., & Liu, Z. (2017). Psychotic-like experiences and correlation with childhood trauma and other socio-demographic factors: A cross-sectional survey in adolescence and early adulthood in China. *Psychiatry Research, 255*, 272-277. doi: 10.1016/j.psychres.2017.03.059

Sun, Y. P., Zhang, B., Dong, Z. J., Yi, M. J., Sun, D. F., & Shi, S. S. (2008). Psychiatric state of college students with a history of childhood sexual abuse. *World J Pediatr, 4*(4), 289-294. doi: 10.1007/s12519-008-0052-4

Thapar, A., Heron, J., Jones, R. B., Owen, M. J., Lewis, G., & Zammit, S. (2012). Trajectories of change in self-reported psychotic-like experiences in childhood and adolescence. *Schizophrenia Research, 140*(1-3), 104-109. doi: 10.1016/j.schres.2012.06.024

Thompson, A., Nelson, B., McNab, C., Simmons, M., Leicester, S., McGorry, P. D., Bechdolf, A., & Yung, A. R. (2010). Psychotic symptoms with sexual content in the "ultra high risk" for psychosis population: frequency and association with sexual trauma. *Psychiatry Res, 177*(1-2), 84-91. doi: 10.1016/j.psychres.2010.02.011

Thompson, J. (2008). Associations between childhood trauma and psychotic-like symptoms among individuals at clinical high risk for psychosis. *Biological Psychiatry, 63*(7, Suppl. S), 120S-120S.

Thompson, J. L., Kelly, M., Kimhy, D., Harkavy-Friedman, J. M., Khan, S., Messinger, J. W., Schobel, S., Goetz, R., Malaspina, D., & Corcoran, C. (2009). Childhood trauma and prodromal symptoms among individuals at clinical high risk for psychosis. *Schizophr Res, 108*(1-3), 176-181. doi: 10.1016/j.schres.2008.12.005

Tomassi, S., Brondino, N., Bonetto, C., Ruggeri, M., Turco, G., Politi, P., & Tosato, S. (2018). The association of childhood trauma with psychotic-like experiences and the role of salivary cortisol levels. An explorative study on healthy subjects from the Italian general population. *Journal of Psychosomatic Research, 109*, 139-140. doi: 10.1016/j.jpsychores.2018.03.155

Torrecilla, P., Gizdic, A., Racioppi, A., Monsonet, M., Kwapil, T. R., & Barrantes-Vidal, N. (2020). STRESS SENSITIZATION AS THE UNDERLYING MECHANISM LINKING CHILDHOOD TRAUMA AND PSYCHOTIC-LIKE SYMPTOMS IN NONCLINICAL YOUNG ADULTS. *Schizophrenia Bulletin, 46*, S232-S232.

Trotta, A., Di Forti, M., Mondelli, V., Dazzan, P., Pariante, C., David, A., Mule, A., Ferraro, L., Formica, I., Murray, R. M., & Fisher, H. L. (2013). Prevalence of bullying victimisation amongst first-episode psychosis patients and unaffected controls. *Schizophrenia research, 150*(1), 169-175.

Valmaggia, L. R., Day, F. L., Kroll, J., Laing, J., Byrne, M., Fusar-Poli, P., & McGuire, P. (2015). Bullying victimisation and paranoid ideation in people at ultra high risk for psychosis. *Schizophrenia Research, 168*(1-2), 68-73. doi: 10.1016/j.schres.2015.08.029

van Os, J., Pries, L.-K., Ten Have, M., de Graaf, R., van Dorsselaer, S., Delespaul, P., Bak, M., Kenis, G., Lin, B. D., Luykx, J. J., Richards, A. L., Akdede, B., Binbay, T., Altınyazar, V., Yalınçetin, B., Gümüş-Akay, G., Cihan, B., Soygür, H., Ulaş, H., Cankurtaran, E. Ş., Kaymak, S. U., Mihaljevic, M. M., Petrovic, S. A., Mirjanic, T., Bernardo, M., Mezquida, G., Amoretti, S., Bobes, J., Saiz, P. A., García-Portilla, M. P., Sanjuan, J., Aguilar, E. J., Santos, J. L., Jiménez-López, E., Arrojo, M., Carracedo, A., López, G., González-Peñas, J., Parellada, M., Maric, N. P., Atbaşoğlu, C., Ucok, A., Alptekin, K., Saka, M. C., Arango, C., O'Donovan, M., Rutten, B. P. F., & Guloksuz, S. (2020). Evidence, and replication thereof, that molecular-genetic and environmental risks for psychosis impact through an affective pathway. *Psychological medicine*, 1-13. doi: 10.1017/S0033291720003748

van Zelst, C., van Nierop, M., van Dam, D. S., Bartels-Velthuis, A. A., Delespaul, P., & null, n. (2015). Associations between Stereotype Awareness, Childhood Trauma and Psychopathology: A Study in People with Psychosis, Their Siblings and Controls. *PLoS ONE, 10*(2), 1-15. doi: 10.1371/journal.pone.0117386

Varese, F., Barkus, E., & Bentall, R. P. (2011). Dissociative and metacognitive factors in hallucination-proneness when controlling for comorbid symptoms. *Cognitive neuropsychiatry, 16*(3), 193-217.

Velikonja, T., Velthorst, E., McClure, M. M., Rutter, S., Calabrese, W. R., Rosell, D., Koenigsberg, H. W., Goodman, M., New, A. S., Hazlett, E. A., & Perez‐Rodriguez, M. M. (2019). Severe childhood trauma and clinical and neurocognitive features in schizotypal personality disorder. *Acta Psychiatrica Scandinavica, 140*(1), 50-64. doi: 10.1111/acps.13032

Velikonja, T., Velthorst, E., Zinberg, J., Cannon, T. D., Cornblatt, B. A., Perkins, D. O., Cadenhead, K. S., Tsuang, M. T., Addington, J., Woods, S. W., McGlashan, T., Mathalon, D. H., Stone, W., Keshavan, M., Seidman, L., & Bearden, C. E. (2021). Childhood trauma and cognitive functioning in individuals at clinical high risk (CHR) for psychosis. *Dev Psychopathol, 33*(1), 53-64. doi: 10.1017/S095457941900155X

Veling, W., Counotte, J., Pot-Kolder, R., van Os, J., & van der Gaag, M. (2016). Childhood trauma, psychosis liability and social stress reactivity: a virtual reality study. *Psychological Medicine, 46*(16), 3339-3348. doi: 10.1017/S0033291716002208

Velthorst, E., Nelson, B., O’Connor, K., Mossaheb, N., de Haan, L., Bruxner, A., Simmons, M. B., Yung, A. R., & Thompson, A. (2013). History of trauma and the association with baseline symptoms in an Ultra-High Risk for psychosis cohort. *Psychiatry Research, 210*(1), 75-81. doi: 10.1016/j.psychres.2013.06.007

Waxman, R., Fenton, M. C., Skodol, A. E., Grant, B. F., & Hasin, D. (2014). Childhood maltreatment and personality disorders in the USA: specificity of effects and the impact of gender. *Personality and mental health, 8*(1), 30-41.

Wigman, J. T. W., van Nierop, M., Vollebergh, W. A. M., Lieb, R., Beesdo-Baum, K., Wittchen, H.-U., & van Os, J. (2012). Evidence that psychotic symptoms are prevalent in disorders of anxiety and depression, impacting on illness onset, risk, and severity--implications for diagnosis and ultra-high risk research. *Schizophrenia bulletin, 38*(2), 247-257.

Wigman, J. T. W., van Winkel, R., Ormel, J., Verhulst, F. C., van Os, J., & Vollebergh, W. A. M. (2012). Early trauma and familial risk in the development of the extended psychosis phenotype in adolescence. *Acta psychiatrica Scandinavica, 126*(4), 266-273.

Wigman, J. T.W., van Winkel, R., Jacobs, N., Wichers, M., Derom, C., Thiery, E., ... & Van Os, J. (2011). A twin study of genetic and environmental determinants of abnormal persistence of psychotic experiences in young adulthood. American Journal of Medical Genetics Part B: Neuropsychiatric Genetics, 156(5), 546-552.

Wigman, J. T. W., van Winkel, R., Raaijmakers, Q. A. W., Ormel, J., Verhulst, F. C., Reijneveld, S. A., van Os, J., & Vollebergh, W. A. M. (2011). Evidence for a persistent, environment-dependent and deteriorating subtype of subclinical psychotic experiences: a 6-year longitudinal general population study. *Psychological medicine, 41*(11), 2317-2329.

Wolke, D., Lereya, S. T., Fisher, H. L., Lewis, G., & Zammit, S. (2014). Bullying in elementary school and psychotic experiences at 18 years: a longitudinal, population-based cohort study. *Psychological Medicine, 44*(10), 2199-2211. doi: 10.1017/S0033291713002912

Yang, X., Wang, D., Liu, S., Liu, G., & Harrison, P. (2020). Trajectories of state anhedonia and recent changes in anhedonia in college students: Associations with other psychiatric syndromes. *Journal of Affective Disorders, 262*, 337-343. doi: 10.1016/j.jad.2019.11.043

Yen, S., Shea, M. T., Battle, C. L., Johnson, D. M., Zlotnick, C., Dolan-Sewell, R., Skodol, A. E., Grilo, C. M., Gunderson, J. G., Sanislow, C. A., Zanarini, M. C., Bender, D. S., Rettew, J. B., & McGlashan, T. H. (2002). Traumatic exposure and posttraumatic stress disorder in borderline, schizotypal, avoidant, and obsessive-compulsive personality disorders: Findings from the collaborative longitudinal personality disorders study. *Journal of Nervous and Mental Disease, 190*(8), 510-518.

Young, M. S., Harford, K., Kinder, B., & Savell, J. K. (2007). The relationship between childhood sexual abuse and adult mental health among undergraduates: victim gender doesn't matter. *Journal of Interpersonal Violence, 22*(10), 1315-1331.
